# Testing an Unconventional Mortality Information Source in the Canton of Geneva Switzerland

**DOI:** 10.5539/gjhs.v6n1p1

**Published:** 2013-09-26

**Authors:** Emmanuel Kabengele Mpinga, Véronique Delley, Emilien Jeannot, Joachim Cohen, Philippe Chastonay, Donna M Wilson

**Affiliations:** 1 Institute of Social and Preventive Medicine Faculty of Medicine, Geneva University and Swiss School of Public Health, Zurich, Switzerland; 2 Cantonal Health Directorate, Geneva, Switzerland; 3 Institute of Social and Preventive Medicine, Faculty of Medicine, Geneva University, Switzerland; 4 End-of-life Care Research Group, Vrije Universiteit Brussel & Ghent University, Belgium; 5 Institute of Social and Preventive Medicine, Faculty of Medicine, Geneva University, Switzerland; 6 University of Alberta, Edmonton, Alberta, Canada

**Keywords:** mortality data, death place, emergency, Switzerland, comparative study

## Abstract

Mortality data are often unavailable, incomplete, and difficult to access for research and other purposes. Gaps in mortality data reports, particularly those detailing place of death, deprive healthcare professionals, decision-makers, and many others of the information that is needed to plan, implement, and evaluate interventions designed for purposes such as to assist people in achieving their preferred death place or reduce hospital utilization. Alternative methods of collecting reliable and valid data on death place may be needed. Our study primarily compared mortality data from a conventional information system (Federal Statistical Office) with mortality data collected using an unconventional system (funeral homes) over a 6-year period (2005-10) for the canton of Geneva Switzerland. Only a small average difference (4.8%) in death incidence was found. Death place data comparisons were also useful. This study suggests that the unconventional data from funeral homes provides reasonably reliable, valid, timely, and useful mortality data.

## 1. Introduction

Death place is increasingly being recognized around the world as having major public health importance (Bruera et al., 2003; Cohen et al., 2007; Teno et al., 2013; Walker et al., 2011). In many countries, however, mortality data are incomplete; with place of death either not recorded or specific enough to be useful (Lawson & Burge, 2010). Data collection has time and resource implications, and mortality data collection is also subject to historical influences, as location of death has not always been of interest. This gap is unfortunate, as this information would be helpful for the implementation of prevention programs, such as accident reduction or avoidance measures. There are many other possible uses for death place information; as health care providers in hospitals and nursing homes, lawyers, insurance companies, public health professionals, and even family members in various contexts (such as those who may attain closure through hearing the exact circumstances of death) could use death place data for health or social services planning, policy-making, and many other reasons (Maddison et al., 2012; Shuman et al., 2011; Teno et al., 2013; Walker et al., 2011). For instance, a trend to home deaths over hospital deaths would raise awareness of the need to increase support for family caregivers.

A rapidly growing body of research on death place is evident, however. This research covers various fields of interest, such as a comparison of actual and preferred place of death (Brogaard et al., 2012; Dunlop & Davies, 1989; Holdsworth & Fisher, 2010), trends in location of death (Houttekier et al., 2011; Hunt et al.. 2001; Wilson et al., 2001; Wilson et al., 2009), determinants of place of death and both predictive or explicative factors for death in select places (Fukui et al., 2011; Gallo et al., 2001, Goodridge et al., 2010; Hong et al., 2011), the impact of home care programs and palliative care services on place of death (Alsirafy et al., 2010; Maddison et al., 2012; Neergarrd et al., 2010; Sessa et al. 1996; Teno et al., 2013; Vadeboncoeur et al., 2010), and the required roles of family members and healthcare professionals for providing care at home or elsewhere (Cavalli, 2002; Neergarrd et al., 2010; Stajduhar & Davies, 1998). More recently, research has been directed to understanding the relationship between place of death, quality of life, and end-of-life social inequalities (Coupland et al., 2011; Mukamel et al., 2012; Shuman et al., 2011; Wright et al., 2010).

Despite this surge of interest in death place, there has been little research examining the quality of death information systems or mortality databases, and the usefulness of these data collection and management systems. Of note is one relatively recent study, conducted by Lawson and Burge (2010), who revealed available Canadian death certificate information on death place was problematic since the recording of a hospital death (since only the death location is noted) masks the extent of community-based end-of-life care. We conducted a study, however, because of the need for an alternative source of information on death place and/or end-of-life care place as these data has not been collected since 1986 by the Swiss Federal Public Health Office. The Swiss Federal Office of Public Health is the equivalent of the Health Ministry in other countries. Some mortality data is still available through the Swiss Federal Statistics Office, with annual reports issued on the number of deaths and the various causes of death. This gap means specific information on place of death in Switzerland is no longer available for normal purposes and for action in times of crisis where there is sudden increased mortality or mortality risk, such as with heat waves or influenza epidemics. This information gap is also the case in some other countries. A European study comparing the information systems of 28 countries revealed that the frequency of publication of mortality data ranged from daily (in Portugal and Spain) to annually (such as in Switzerland, although on a limited or minimal basis), and that data on some variables were not always available (Kanieff et al., 2010). For instance, in Germany and Portugal (and Switzerland) routinely collected data did not include any information on place of death (Kanieff et al., 2010).

## 2. Method

This study was designed to compare mortality data (specifically incidence of death) from the demographic information system of the Federal Statistical Office and an unconventional system (funeral homes) over a 6-year period (2005-10) in the canton (i.e. state) of Geneva Switzerland. The unconventional system was initiated after the 2003 heat wave when it became evident that a rapid means of detecting excess deaths was needed. Death place data comparisons were also undertaken, using available 1970-1986 Federal Statistics Office data (with 1970 and 1985 data reported), 2000 Cantonal Statistics Office data, and 2010 Geneva Observatory death data (although an estimate for 12 months is provided from 6 months of data). The study was aimed at testing the reliability of this new unconventional system, and identifying the strengths and weaknesses of it, along with conditions for success.

### 2.1 Sources of Data

**Figure 1:**
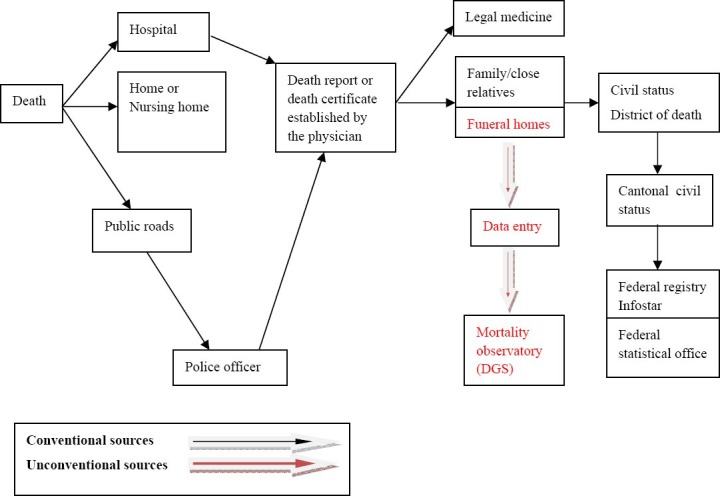
Mortality data collection procedures in the canton of Geneva Switzerland

Data from the conventional sources were derived from state registries maintained by the Federal Statistical Office. Post-mortem procedure requires a death report from the physician or the police, the death certificate from the attending physician, the death certificate’s transfer to the family and funeral home, the encoding of data relating to various information on the certificate in an electronic file at the district level, and the transfer of these data to a centralized computerized national registry (i.e. Infostar) in Berne Switzerland (so as to make this data available to the Federal Statistical Office). This procedure, illustrated in Figure 1, requires an average of one year to complete, with this time frame in part due to the extra time to code certified causes of death into ICD-10 codes. Access to this data for the specified time and place (Geneva canton) was provided to the research team.

Data from the unconventional sources were obtained from an ad hoc newly created mortality observatory of the Geneva canton. This observatory was implemented in the aftermath of the 2003 heat wave, with contributors being the Institute of Social and Preventive Medicine (Institut de médecine sociale et preventive), the Geriatric Policlinic (Policlinique de gériatrie), the Health Directorate (Direction de la santé), and local funeral homes. In the Geneva canton, three companies provide funeral services. The data collected were based on the death certificate issued by the attending physician or police officer in charge, a copy of which was delivered to the funeral home. By mutual agreement, each funeral home sent daily faxes with detailed information on these registered deaths to the observatory officer (the identities of the deceased were not disclosed). The officer in charge of registration at the observatory entered the data in an Excel file recording the following parameters, date and hour of death, name of the funeral home in charge of the funeral arrangements, year of birth, gender, place of residence, and place of death. The officer then entered the data into the mortality database of the Health Directorate (Direction Générale de la Santé). On a weekly basis, a senior officer at the Health Directorate checked the Excel file using the faxes’data as control. The entire procedure is illustrated in Figure I (in red).

## 3. Results

Table I illustrates the comparative findings of the two information sources on death incidence in Geneva canton from 2005 through 2010. There was an average discrepancy of 4.8% across all years, ranging from 2.4% to 8.4% each year. Table II illustrates death place comparisons, using available 1970-1986 Federal Statistics Office data (with 1970 and 1985 data reported), 2000 Cantonal Statistics Office data, and 2010 Geneva Observatory death data (although an estimate for 12 months is provided from 6 months of data). The following trends were revealed,

(a) a reduction in the proportion of deaths occurring at home,
(b) an increase in the proportion of deaths occurring in medico-social institutions such as nursing homes, and
(c) relatively minor changes overall in the proportion of deaths from 1985 to 2010 occurring in hospitals.

**Table 1:** Death incidence comparison using conventional and unconventional information systems, Geneva Canton, 2005-2010

	2005	2006	2007	2008	2009	2010
Total number using unconventional sources	**2940**	**2940**	**2846**	**2843**	**3025**	**3024**
Total number using conventional sources	3054	3012	3086	3065	3140	3128
Difference (number)	-114	-72	-240	-222	-115	-104
Difference (%)	3.8	2.4	8.4	7.8	3.6	3.3

**Table 2:** Death place over time and location, Geneva Canton, 1970-2010

Period	Total number	Home Deaths		Deaths in Hospital		Medico-social institutions and other nursing homes	
1970	2,818	779	27.6%	2,048	72.6%	129	4.5%
1985	3,138	740	23.5%	1,787	56.9%	611	19.4%
2000	3,124	618	19.7%	1,585	50.7%	821	26.2%
2010 e	3,024	486	16.07%	1,712	56.6%	826	27.3 %

Data Sources:

1970-1985, Federal Statistical Office Mortality Database

2000, Cantonal Statistical Office Data

2010, Geneva Observatory estimates from data for first 6 months of the year

## 4. Discussion

Our study results show that the mortality data, and specifically the number or incidence of deaths, collected from the ad hoc mortality observatory (the unconventional funeral homes information source) was relatively similar to the conventional information. The minor discrepancies between data sources in terms of death incidences can be accounted for by the following observations, (a) some funerals will not be handled by the three major funeral homes in Geneva, (b) data entry errors, and (c) some Geneva canton inhabitants die outside of the Geneva canton, either elsewhere in Switzerland or abroad. For example, it is known that during the last 6 months of 2004, approximately 23 deaths out of a mean monthly total of 245 (9.3%) that were recorded in Geneva canton actually occurred outside the canton (Delley, 2005). As such, this study indicates funeral home data could provide a more rapid source of reasonably accurate information when this information is needed. Public health crises that result in a sudden increase in the death rate can potentially be quickly revealed and monitored by such information in Geneva and perhaps elsewhere, thus allowing quick and relevant public health and implementation of preventive programs related to specific mortality patterns.

The comparative data on place of death were also revealing. The 2010 data collected by the Observatory from funeral homes was in line with what could be expected from conventional data sources, although with death location trends over time needing to be taken into account. This suggests that the ad hoc mortality Observatory is a good alternative method to collect place of death data, a matter of considerable relevance as the Swiss Federal Public Health Office no longer collects such information.

The concordance of our data with the data collected from conventional information sources allows us to draw two primary conclusions on the representativeness and sensitivity of data collected using the unconventional information system. The data representativeness is largely assured because the three funeral home companies (i.e. Entreprise Murrit, Pompes funèbres Générales de Genève, and Pompes funèbres officielles) have almost exclusive control of the Geneva canton funeral market (Delley, 2005). Data sensitivity is indicated by the fluctuations in data over time.

It should also be noted that other studies have confirmed the validity of mortality data based on funeral homes, coffin sales, and religious ceremonies in Malawi and Ethiopia - where official statistics are lacking—for the monitoring of mortality rates due to HIV/AIDS (Araya et al., 2011; Mwangomba et al., 2010).

The question of data reliability must be addressed by considering the competence of the involved institutions and the mechanisms implemented for data collection, transmission, and analysis of importance to consider. The following indicate considerable data reliability through the unconventional information system:

(a) legal and technical competence, as the system was implemented on the initiative of the Cantonal Health Directorate in collaboration with the Institute of Preventive and Social Medicine at the Medical Faculty of the Geneva University, and the funeral homes;
(b) scientific competence, as assured by the Institute of Preventive and Social Medicine, which was primarily in charge of designing, testing, and the critical analysis of the collected data; and
(c) data coverage and representativeness was assured by the funeral homes, since these have information on almost all deaths occurring on cantonal territory.

As such, one could indicate that the collected data were reasonably reliable. This raises the key question of the usefulness of such a system; particularly in times of health crises or emergency situations. Any system’s usefulness is largely dependent on its reactivity and time-saving quality. Whereas Swiss mortality data from conventional systems is only available on an annual basis and does not take into account place of death now, mortality numbers through the Observatory is available within 4 to 5 days, and with the place of death data also available for additional consideration. This time period could be shortened further in crisis situations.

Also worthy of highlighting is the unconventional information system’s simplicity. This simplicity, and thus the relatively low cost to set up and maintain it, is largely attributable to the fact that the data collection, centralization, and diffusion circuits are the shortest and easiest possible. More specifically, the implemented procedures did not require major human, computer, or other logistic resources. The data were easily obtained by fax and easily entered in an Excel sheet, and were limited to a small number of variables (i.e. name of funeral home, gender, year of birth, date and hour of death, place of death, and town/village). Where the conventional system relies on six steps, many of which involve formal or legislated processes, the Observatory system requires on only four in total, starting with the attending physician’s completion of the death certificate. These factors maximally limit the loss of data, data reporting lags, and data inaccuracies. The Observatory system could also be described as comprehensible and accessible to all (Dufour et al., 2006). Also worth mentioning is the unconventional system’s promise for achieving the objectives for which it was designed--specifically, providing Geneva with mortality data within a short time frame so as to be a quick alert system for monitoring mortality figures in crisis or emergency situations.

It should also be stressed that the unconventional system is cost-effective, as it is based on the voluntary and gratuitous participation of all involved participants, including the funeral home operators who kindly agreed to participate without any financial compensation. However, this lack of financial compensation must be considered as one of the system’s weaknesses; as any party, in the absence of any legal framework or well-understood incentive for participation, could delay or block the data collection. These difficulties are well-known in the field of collaborative work, as Prete (2008) suggested.

However, this unconventional system was readily implemented in this Swiss canton. The contributing factors for this success are notable. All involved actors, at the project’s initiation, mutually perceived a need for a tool using mortality data so that the data would function as an alert system. Recommendations highlighting this need were made at a 2004 conference designed to reflect upon the consequences of the 2003 European heat wave. One such recommendation was the implementation of a simple mechanism to collect mortality data in the Geneva canton (Colloque Canicule, 2004). A second condition of success is technical in nature as it refers to the limited number of funeral home companies in the Geneva area (three in total), their control of almost the entire funeral market, and their willingness to participate in the project. Lastly, the willingness of the various institutional authorities to implement such an Observatory was critical. It is noteworthy that this institutional authority cooperation does not necessarily translate into improved political and social visibility; as except for our study, there has been no further analysis of the collected data. Unless the data are more fully utilized, the involved actors may find little justification for their ongoing involvement in this project. However, the motivation of the actors for organizing this system and its potential impact have already been considered success factors for constructing and continuing this information system (Mpinga et al., 2003).

Regardless, one major limitation of this specific unconventional data system is the limited number of mortality variables provided and thus recorded. As cause of death data were not obtained, this reduces the Observatory’s usefulness for epidemiological and other studies. This limitation was due to regulatory constraints (i.e. a 1992 federal law on data protection did not permit the sharing of cause of death data) over technical issues, such as the system’s inability to collect such data.

Another limitation is that this study suffered from a short observation period (2005-2010). This period was too brief to test the observatory system in vivo - such as in a real humanitarian crisis situation. In addition, it missed official data on places of death for 2010 comparative purposes. This lack of official standard reference data restricted us from conducting a more useful comparison between the two systems regarding place of death data accuracy and completeness.

However, many studies on place of death lack methodological rigor (Mpinga et al., 2003). The quality and reliability of the data presented in past published studies is thus of some concern, as is the data source (i.e. cancer registry, hospital statistics, etc.); all of which have hardly been evaluated (Mpinga et al., 2003). At the very least, our study was an attempt to compare mortality data across different mortality data information systems; with an unconventional system found to have reasonably reliable data. The greatest promise from this unconventional system is data accessibility for public health decision-makers in times of crisis, and also for other health policy decisions, such as when there may be a need to construct additional nursing homes or initiate road accident prevention programs.

## 5. Conclusion

Our study was designed in part to emphasize alternative models for collecting mortality data, particularly when the conventional information model has information gaps or hazards such that it reduces the ability to respond quickly in times of crisis. The unconventional information system (that was based on funeral home data) was shown to provide reasonably reliable, valid, and potentially useful mortality data. Although unconventional information systems cannot make the same claims of completeness as conventional systems, their “real-time” data should render them useful to decision-makers and opinion-leaders in periods of emergency, crisis, or disaster. They may also fill gaps in the collection, analysis, and publication of mortality data from conventional systems, such as information gaps on place of death. Having data readily available would address the considerable concern over the normal long delays in the publication or sharing of mortality data reports. Data delays deprive public health professionals, decision-makers, and opinion leaders of the information they need in order to manage public opinion, plan interventions, and implement short- to longer-term health action plans. Our study and these issues highlight the need for additional research on alternative unconventional information sources that would provide decision-makers and others with timely access to the information they need. Timely information is needed to enable them to understand the magnitude of a crisis, problem, or trend; assess and then determine what responses are the best under the circumstances, and then undertake necessary actions.
